# Type XII collagen is elevated in serum from patients with solid tumors: a non-invasive biomarker of activated fibroblasts

**DOI:** 10.1007/s10238-024-01431-y

**Published:** 2024-07-24

**Authors:** Marina Crespo-Bravo, Annika Hettich, Jeppe Thorlacius-Ussing, Thomas R Cox, Morten A. Karsdal, Nicholas Willumsen

**Affiliations:** 1 Nordic Bioscience A/S, 2730 Herlev, Denmark; 2 Department of Biomedical Sciences, University of Copenhagen, 2200 Copenhagen, Denmark; 3 Matrix and Metastasis Lab, Cancer Ecosystems Program, Garvan Institute of Medical Research and The Kinghorn Cancer Centre, Darlinghurst, NSW Australia; 4 School of Clinical Medicine, UNSW Medicine & Health, UNSW Sydney, Sydney, NSW Australia

**Keywords:** Type XII collagen, ECM, CAFs, FACITs, Non-invasive biomarker, Metastasis

## Abstract

Understanding the tumor microenvironment (TME) and extracellular matrix (ECM) is crucial in cancer research due to their impact on tumor progression. Collagens, major ECM components, regulate cell signaling and behavior. Of the 28 reported collagens, type XII collagen is known to be vital for ECM organization. Over-produced by cancer-associated fibroblasts (CAFs), its upregulation correlates with poor survival in various cancers. This study aimed to develop an ELISA for quantifying circulating type XII collagen as a cancer biomarker. A specific ELISA targeting the C-terminal of type XII collagen was developed and used to analyze serum samples from cancer patients (*n *= 203) and healthy controls (*n* = 33). Additionally, type XII collagen expression was assessed in CAFs and normal fibroblasts (NFs) from different tissues, both under TGF-β stimulated and non-stimulated conditions. The nordicPRO-C12 ELISA demonstrated robustness and specificity for type XII collagen. PRO-C12 levels were significantly elevated in patients with various cancers compared to healthy controls and effectively distinguished between cancer patients and controls. Findings were validated using gene expression data. Furthermore, Western blot analysis revealed increased type XII collagen expression in both CAFs and NFs upon TGF-β1 stimulation, suggesting a potential role of TGF-β1 in modulating the expression of type XII collagen in cancerous and normal tissue microenvironments. This study unveils a promising avenue for harnessing PRO-C12 as a non-invasive serum biomarker, enabling the quantification of type XII collagen fragments in cancer patients. Further investigations are warranted to explore the potential of PRO-C12 across different cancer types and disease stages, shedding light on its multifaceted role in cancer development.

**Supplementary Information:**

The online version contains supplementary material available at 10.1007/s10238-024-01431-y.

## Introduction

Cancer remains a global health challenge, with its incidence steadily rising worldwide [1]. The complexity of cancer, together with the lack of efficient diagnostic and prognostic tools, underscores the urgent need for innovative approaches to enhance early detection and prognosis. Traditional diagnostic methods often pose challenges, including invasiveness and limitations in providing comprehensive information. In this context, the development on non-invasive biomarkers emerges as a promising avenue, offering the potential to revolutionize cancer diagnosis and monitoring. In recent years, the tumor microenvironment (TME) consisting of tumor cells, stromal cells, immune cells and extracellular matrix (ECM) has garnered significant attention due to its critical role in tumor progression and impact on treatment outcomes. Numerous studies have revealed variations in the ECM composition between the different cancer types which highlights the pivotal role of the TME in cancer development and underscores the need for targeted therapeutic strategies that consider the intricacies of this microenvironment [2–5]. Collagens stand as the predominant components of the ECM, shaping the structural framework of tissues and organs. 28 different types of collagens have been identified to date, providing essential structural support, tensile strength, and elasticity to tissues, influencing cell behavior and multiple physiological processes. In addition to acting individually, the different collagens interact with one another to form large polymeric supramolecular structures which also provide cues to resident cells. Beyond their structural role, collagens actively participate in cell signaling, migration and differentiation [6, 7]. Moreover, collagen fragments originated from excessive remodeling of the ECM during tumor progression have been previously proposed as cancer biomarkers [8–15]. For instance, high levels of the fibrillar type III collagen pro-peptides in circulation reflect tumor fibrosis activity and predicted poor prognosis in patients with different solid cancer types [15–20].

Although less abundant than fibrillar collagens, FACIT collagens (fibril associated collagens with interrupted triple-helices) including type IX, XII, XIV, XIX, XX, XXI and XXII are associated with fibrillar collagens and act as molecular bridges, playing a crucial role in organizing and maintaining the stability of ECM. FACITs have multiple triple-helical domain separated by non-triple-helical domains, and unlike fibrillar collagens, they do not undergo proteolytic processing from a larger precursor form [21, 22]. In particular, type XII collagen was first discovered by cDNA cloning with partial homology to the α1 chain of type IX collagen associated with type I collagen fibrils [23–25]. Type XII collagen is composed of two small collagenous domains (COL1 and COL2) separated by non-collagenous domains (NC1 and NC2), and a large N-terminal domain (NC3) [26, 27]. Splicing in the NC3 domain originate two isoforms, XIIA (large isoform) and XIIB (small isoform), and therefore type XII collagen molecules can assemble as homotrimers or as a combination of XIIA and XIIB heterotrimers and their expression depends on tissue localization and developmental stage [24, 28, 29]. Increased expression of type XII collagen has been reported upon mechanical stress but further research is warranted to explore the molecular mechanisms in this process [30–32]. Due to its molecular structure and tissue distribution, type XII collagen seems to play a role in type I collagen fibrillogenesis, fibril organization and interactions with other ECM molecules [21, 33–35]. Additionally, type XII collagen is expressed in bone, muscle and tendons and regulates tissue regeneration and communication between cells during development [36–39]. Moreover, mutations in the *COL12A1* are associated with myopathic type Ehlers-Danlos syndrome (mEDS) characterized by muscle weakness, distal joint hypermobility and delayed tendon reflexes [40]. In recent years, multiple studies have reported that type XII is involved in cell migration and invasion and tumor growth [41–43]. *COL12A1* overexpression has been detected and was predictive of poor prognosis in multiple cancer types including colorectal (CRC), gastric, breast cancer, renal, ovarian and pancreatic cancer [41, 43–54]. Further, in patients with breast cancer high expression of *COL12A1* predicted poor response to immunotherapy treatment [50, 55]. Interestingly, a recent study demonstrated that type XII collagen secreted by cancer associated fibroblasts (CAFs) modifies type I collagen organization surrounding the tumor and stablishing a pro-metastatic environment for cancer cells dissemination and proposed type XII as a tool to identify patients that are more prone to breast cancer relapse [49]. In this study, we developed and validated enzyme-linked immunosorbent assay (ELISA) to measure the C-terminus of type XII collagen (PRO-C12) that could detect both XIIA and XIIB isoforms in serum to evaluate its biological relevance. We found increased levels of PRO-C12 in serum from patients with different cancer types and additionally, PRO-C12 could discriminate between cancer patients and healthy controls underlaying its biomarker potential.

## Materials and methods

### PRO-C12 competitive ELISA development

The 10 amino acid peptide ^3053^YNGQGYPGSG^3063^ found in the C-terminus of type XII collagen (UniProtKB: Q99715) was purchased from Genscript (Piscataway, NJ, USA) and used for immunization. This sequence was linked to the immunogenic peptide (KLH-CGG-YNGQGYPGSG) by cross-linking to Keyhole Limpet Hemocyanin (KLH) carrier protein using sulfosuccinimidyl 4-(*N*-maleimidomethyl) cyclohexane-1-carboxylate (SMCC, Thermo Scientific, Waltham, MA, USA, cat. No. 22322). Glycine and cysteine residues were added at the N-terminal end of the sequence for proper binding of the peptide to the carrier protein. Monoclonal antibodies were produced by immunizing six-week-old Balb/C mice with subcutaneous injection of 200 uL emulsified antigen containing 100 ug of immunogenic peptide mixed with Sigma Adjuvant System (Sigma cat. No. S6322). Immunizations were administered at 2-week intervals until stable titers of sera were achieved. The mouse exhibiting the highest titer was given a four-week rest period before receiving a booster dose of 100 ug immunogenic peptide in 100 uL 0.9% NaCl solution intravenously. Hybridoma cells were generated by fusing spleen cells with SP2/0 myeloma cells and the hybridoma cells were then cultured in 96-well microtiter plates. Limited dilution was employed to ensure the monoclonal growth. The selection of the antibody clone targeting the epitope of interest was chosen based on a preliminary competitive ELISA assessing reactivity towards the selection peptide (YNGQGYPGSG), a truncated peptide (YNGQGYPGS), an elongated peptide (YNGQGYPGSGA) and a deselection peptide (PGLPGYPGSP) corresponding to type IV collagen alpha 4 chain with a sequence closely resembling that of the selection peptide that could potentially compete for antibody binding. Subsequently, the monoclonal antibodies were purified using protein-G-columns following the manufacturer’s guidelines (GE Healthcare Life Sciences, Little Chalfont, UK, cat. No. 17-0404-01). To complete the process, the purified antibodies were labeled with horseradish peroxidase (HRP) using a peroxidase labeling kit (Roche Diagnostics GmbH, Mannheim, Germany, cat. no. 11829696001).

### PRO-C12 ELISA protocol

The ELISA underwent multiple optimizations including the selection of assay buffer, incubation duration and temperature and antibody and peptide concentration. The final PRO-C12 protocol included the following steps: a 96-well streptavidin-coated ELISA plate was coated with 100 uL/well of 5 ng/mL biotinylated YNGQGYPGSG peptide in assay buffer (50 mM PBS, 1% BSA (w/v), 0.018% bronidox (v/v), 0.1% Tween-20 (w/v), 4 g/L NaCl, pH 7.4) and incubated for 30 min at 20 °C with shaking at 300 RPM. After 5 washes with washing buffer (25 mM Tris, 50 mM NaCl, pH 7.2), 20 uL/well of the sample diluted 1:2 in assay buffer was added in duplicates, followed by 100 uL/well of 25 ng/mL HRP-labelled monoclonal antibody in assay buffer. The mixture was incubated for 20 h at 4 °C with shaking at 300 RPM. Following a second washing cycle, 100 uL/well of 3,3’,5,5’-Tetramethylbenzidine (TMB) was added and incubated for 15 min in darkness at 20 °C with shaking at 300 RPM. The reaction was quenched by adding 100 uL/well of 1% H_2_SO_4_ (v/v). Absorbance was measured at 450 nm with 650 nm as the reference. For the generation of the standard curve, 20 uL/well of IRGPPGPPGYCDSSQCASIPYNGQGYPGSG 100 ng/mL peptide, serially diluted twofold, was added to the appropriate wells, and a four-parametric logistic regression model (4PL) was used for curve fitting. Each plate included five quality control samples, comprising two human serum, one sheep serum, one pig serum and one human serum spiked with standard peptide.

### Technical validation of the PRO-C12 ELISA

Antibody specificity evaluation involved testing of the standard peptide (IRGPPGPPGYCDSSQCASIPYNGQGYPGSG) assessed in twofold dilution series. Additionally, an elongated version (YNGQGYPGSGA) and a truncated peptide (YNGQGYPGS) of the PRO-C12 epitope were tested as well as a deselection peptide (PGLPGYPGSP). To assess linearity of dilution, twofold dilution of human serum samples were measured, and the percentage recovery of the measured concentration relative to the predicted concentration was calculated. Accuracy was evaluated by spiking a known quantity of the standard peptide into human serum samples or by spiking one human serum sample into another at different ratios (100:0, 75:25, 50:50, 25:75, or 0:100), followed by calculating the percentage recovery of the spiked sample relative to the non-spiked sample. The impact of interfering substances commonly found in serum including hemoglobin, lipids and biotin was assessed by spiking human serum samples with known quantities of these substances (hemoglobin low = 2.5 mg/mL, high = 5 mg/mL; lipids low = 1.5 mg/mL, high = 5 mg/mL; and biotin low = 5 ng/mL, high = 100 ng/mL), and the recovery relative to the non-spiked sample was calculated. Assay variation was tested through ten independent runs using ten quality control samples in double determinations. Intra-assay variation was calculated as the mean coefficient of variance (CV%) between double determinations in each run (CV% < 10), while inter-assay variation was calculated as the mean CV% across all ten runs (CV% < 15). The lower (LLMR) and upper (ULMR) limits of the measurement range were determined across the ten independent runs, representing the boundaries of the linear range observed in the standard curve. The lower limit of detection (LLOD) was calculated as the mean interpolated concentration of 60 blank samples in single determinations containing only assay buffer plus three standard deviations while the upper limit of detection (ULOD) was calculated as the mean interpolated concentration of the standard peptide corresponding to the highest concentration of the standard curve minus three standard deviations. The lower limit of quantification (LLOQ) and upper limit of quantification (ULOQ) were determined based on the minimum concentrations at which the average coefficient of variation (CV%) for serum samples remained below 20%. Analyte stability was evaluated for three human serum samples incubated at either 4, 18 and 36 °C after 4 hours, 24 hours and 48 hours of storage and the percentage recovery of the incubated samples relative to the corresponding control sample kept at − 20 °C was calculated. Freeze-thaw stability was assessed by repeatedly freezing and thawing human serum samples for up to five rounds, and the percentage recovery of the samples relative to the corresponding control samples subjected to a single freeze-thaw round was calculated.

### Fibroblasts cell culture – Scar-In-A-Jar

The Scar-In-A-Jar (SiaJ) methodology used in this study was described previously [56, 57]. Native human quiescent normal fibroblasts (NFs) and cancer associated fibroblasts (CAFs) from pancreas, breast and lung were purchased from Vitro biopharma (cat# SC00A5, cat# CAF06, cat# CAF07-AD, cat# CAF08, Golden, CO, USA), and Lonza (cat# CC-2512, Morrisville, NC, USA). Fibroblasts were cultured in flasks coated with 5 µg/cm2 type I collagen purified from rat tail tendon (cat# P8188, Innoprot, Derio, Biscay, Spain). When the confluency reached 90%, 30,000 fibroblasts per well were seeded in 48-well plates, and the medium was replaced with Gibco DMEM.

 + GlutaMAX (cat# 31966047, Thermo Fisher Scientific, Waltham, MA, USA) supplemented with 10% fetal bovine serum (FBS) (cat# F7524, Sigma Aldrich, St. Louis, MO, USA) and 1% penicillin/streptomycin (P/S) (cat# P4333, Sigma Aldrich, USA). After 24 hours, the culture medium was changed to ficoll medium (50% DMEM + GlutaMAX supplemented with 0.4% FBS, 1% P/S, and 50% 70 and 400 kDa Ficoll (cat#17031050 and cat#17030050, Cytvia, Marlborough, MA, USA) dissolved in DMEM + GlutaMAX supplemented with 0.4% FBS, 1% P/S) supplemented with 0.05 mg/ml L-ascorbic acid. Fibroblasts were either treated with 1 ng/ml tgf-β1 (cat# 7754-BH/CF, Bio-techne, Minneapolis, MN, USA) or not treated. Cell supernatant was collected and preserved at − 20 °C for analysis on days 3, 6, 9 and 12. After removal of cell supernatant, new ficoll medium with or without tgf-β1 was added to the respective wells.

### Western blot of SiaJ supernatant

SiaJ Supernatant on day 9 was electro-phoresed on a NuPAGE 4–12% Bis–Tris gel (Invitrogen, Carlsbad, CA, US) under reducing conditions using NuPAGE® MES SDS running buffer (Invitrogen, Carlsbad, CA, US). The proteins from the polyacrylamide gel were then transferred onto an iBlot® nitrocellulose membrane (Life Technologies, Bengaluru, India) using the iBlot® Dry blotting system (Life Technologies, Carlsbad, CA, US). Following this, the membrane was blocked for 1 hour with 5% skim milk (Sigma–Aldrich, St. Louis, MO, USA) in TBST (Tris-Buffered Saline (TBS) with 0.1% Tween-20). The membrane was incubated overnight at 4 °C with type XII collagen monoclonal antibody (PRO-C12 ELISA antibody) and GAPDH loading Control monoclonal antibody (GA1R) (Cat # MA5-15738, Thermo Fisher Scientific, Waltham, MA, USA). Subsequently, the membrane was washed in TBST three times for 10 minutes and then incubated with the secondary peroxidase-conjugated antibody (1:5000, Jackson ImmunoResearch, West Grove, PA, US) for 1 hour. After another wash in TBST, the membrane was incubated for 3 minutes with Clarity Max Western ECL substrate (Cat # 1705062, Bio-Rad Laboratories Inc, Waltham, MA, USA). The bands were visualized using the C-DiGit™ Blot Scanner (LI-COR Biosciences, Lincoln, NE, USA). ImageLab software version 6.1 (Bio-Rad) was employed for image acquisition.

### Patient samples

The cohort consisted of serum samples obtained from 203 patients diagnosed with cancer and 33 healthy controls. This cohort was categorized into 11 groups, each comprising patients with specific cancer types, including bladder (19), breast (19), colorectal (19), head and neck (20), kidney (16), lung (17), ovarian (20), pancreatic (17), prostate (19), gastric cancer (17) or melanoma (20) and 33 age-matched healthy controls. The serum samples from cancer patients and healthy controls were sourced from Proteogenex (Los Angeles, CA, USA) and BioIVT (Westbury, NY, USA), respectively, and were stored at − 80 °C before analysis. A detailed overview of the cohort characteristics is available in Table 1. According to the vendors, the sample collection process received approval from an Institutional Review Board or Independent Ethical Committee, and patients provided informed consent at the Russian Oncological Research Centre n.a. Blokhin RAMS (PG-ONC 2003/1) and Western Institutional Review Board, Inc. (WIRB® Protocol #20161665). All investigations adhered to the principles outlined in the Helsinki Declaration.

**Table 1:** Demographics of the Proteogenex cohort

Characteristic	Cancer, N = 203	Healthy, N = 33
Diagnosis, *n* (%)		
Bladder cancer	19 (9.4)	–
Breast cancer	19 (9.4)	–
Colorectal cancer	19 (9.4)	–
Head & neck cancer	20 (9.9)	–
Kidney cancer	16 (7.9)	–
Lung cancer	17 (8.4)	–
Melanoma	20 (9.9)	–
Ovarian cancer	20 (9.9)	–
Pancreatic cancer	17 (8.4)	–
Prostate cancer	19 (9.4)	–
Gastric cancer	17 (8.4)	–
Healthy donors	–	33 (100)
Stages, *n* (%)		
I	7 (3.5)	–
II	44 (21.8)	–
III	84 (41.6)	–
IV	68 (33.7)	–
Age, Mean (SD)	60 (11)	57 (6)
Sex, *n* (%)		
Male	107 (53)	16 (70)
Female	96 (47.5)	7 (30)

### Analysis of Publicly Available Genomics Databases

The TCGA (https://www.cancer.gov/tcga) and GTEx (https://www.gtexportal.org/) datasets were accessed through the UCSC Xena browser (http://xena.ucsc.edu/). Within the Xena browser, we focused on the TCGA TARGET GTEx combined cohort, specifically filtering down to TCGA and GTEx patients. For *COL12A1* gene expression analysis, we used RSEM expected count (DESeq2 normalized) UCSC Toil RNA-seq Recomputed data. The processing and normalization details of this data have been previously described [58–61]. Two comparison groups were created by combining the GTEx normal data with TCGA normal data, facilitating comparison with the TCGA primary tumor data.

### Statistics

Differences in the PRO-C12 levels among groups were analyzed using Kruskal-Wallis test corrected for multiple comparisons using Dunn’s test. Differences in *COL12A1* gene expression between healthy and cancer tissue was analyzed using unpaired t-test. Diagnostic accuracy was tested by the AUROC curve. Sensitivity and specificity were determined at the estimated optimal cut-off value according to the Youden index. A *p* value below 0.05 was considered significant. In our Western blot analysis, type XII collagen was quantified by measuring the band intensity using ImageJ software (version 1.54g) and then normalized to the loading control, GAPDH. Asterisks indicate the following significance levels: *p < 0.05; **p < 0.01; ***p < 0.001; ****p < 0.0001. When doing multiple comparisons tests, multiplicity adjusted *p*-values are reported. Statistical analysis and graphs were done in GraphPad Prism (version 10.1.1 for Windows, GraphPad Software, San Diego, CA, USA, www.graphpad.com), MedCalc (MedCalc Statistical Software version 22.003 (MedCalc Software Ltd, Ostend, Belgium; https://www.medcalc.org; 2023) and R version 4.3.1 (R Core Team (2023), R Foundation for Statistical Computing, Vienna, Austria, https://www.R-project.org.

## Results

### PRO-C12 ELISA development and validation

**Fig. 1:**
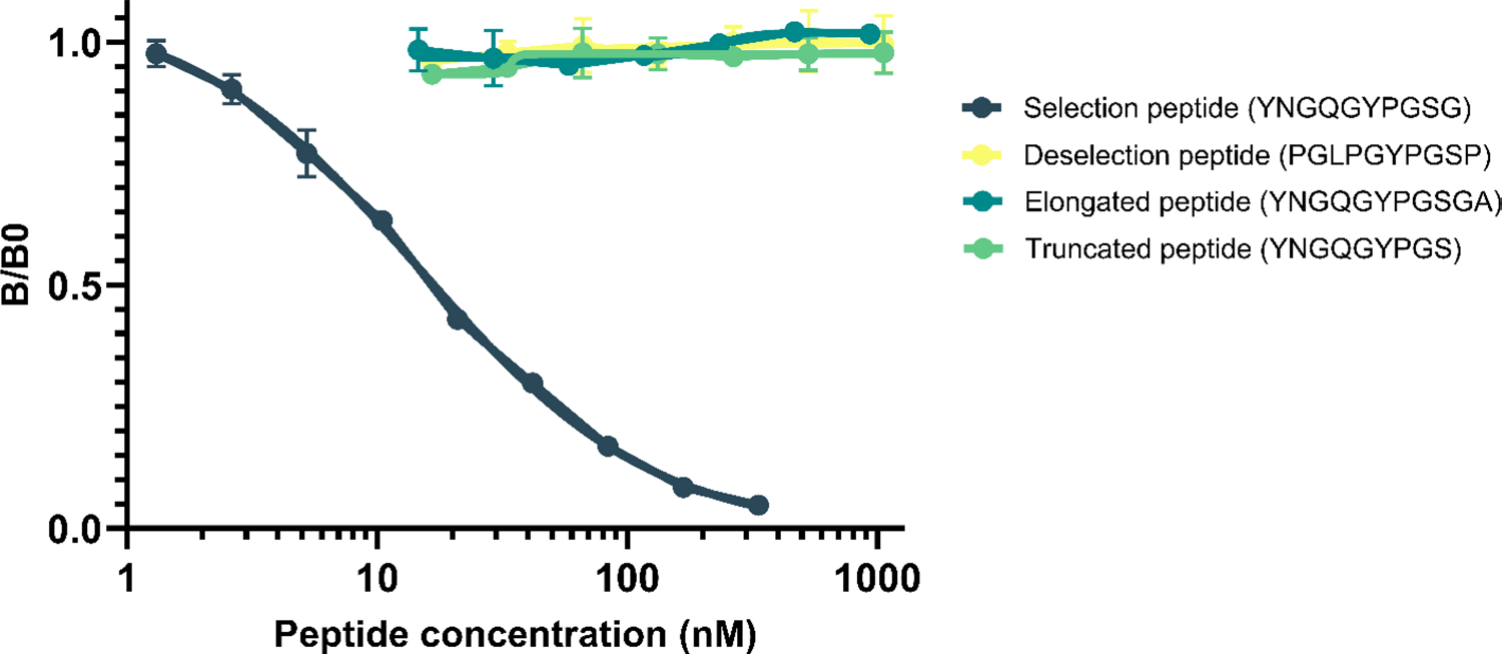
Specificity of the PRO-C12 assay. Inhibition curve for the standard peptide (IRGPPGPPGYCDSSQCASIPYNGQGYPGSG), deselection peptide (PGLPGYPGSP), elongated peptide (YNGQGYPGSGA) and truncated peptide (YNGQGYPGS). Peptides were diluted twofold

The final PRO-C12 ELISA conditions were selected based on the ability to achieve optimal sensitivity in human serum samples while adhering to specified technical criteria that included determining the optimal incubation time and temperature, selecting the most suitable assay buffer, and adjusting concentrations of kit components. Specificity of the PRO-C12 ELISA was evaluated based on the ability of peptides to compete for binding to the monoclonal antibody. The tested peptides included the standard peptide (IRGPPGPPGYCDSSQCASIPYNGQGYPGSG), an elongated peptide (YNGQGYPGSGA), a truncated peptide (YNGQGYPGS_) and a deselection peptide (PGLPGYPGSP). Notably, only the standard peptide exhibited a dose-dependent inhibition of the signal, as depicted in Figure 1. Overall, these results indicate the assay’s specificity for the YNGQGYPGSG epitope at the C-terminus of type XII collagen. Technical aspects of the PRO-C12 assay are summarized in Table 2. Linearity of dilution and parallelism to the standard curve was confirmed when serum samples were diluted at a ratio of 1:2, with an average recovery of 92 %. Accuracy testing through spiking recovery tests demonstrated a good recovery of the standard peptide in human serum, achieving an average recovery of 90 %. Similarly, matrix-in-matrix spiking which involves the addition of one human serum sample into another human serum sample, yielded an average recovery rate of 97 %. Even at high concentrations of commonly interfering substances in the serum such as hemoglobin, lipids and biotin, recovery rates remained within the 15% range. Assay variation, both inter- and intra-assay was 12 and 5% respectively. Analyte stability, conducted over a span of up to 48 at either 4, 20 or 37 °C showed recoveries within the 15% range. Furthermore, stability after five freeze-thaw cycles demonstrated an average recovery rate of 98%. The measurement range was 1.9 ng/mL–100 ng/mL, with a lower limit of detection of 1.29 ng/mL.

**Table 2:** Summary of the technical validation of PRO-C12

Assay parameter	Result
ELISA format	Competitive ELISA with TMB
Intended matrix (MRD)	Human serum (1 + 1)
Incubation buffer	50mM PBS-BTB, 4g/L NaCl
Measurement range (LLOQ-ULOQ)	1.29–100 ng/mL uncorrected 2.58–200 ng/mL corrected
LLOB	1.29 ng/mL
Mean slope	1.3
Mean IC25	2.76 ng/mL
Mean IC50	6.42 ng/mL
Mean IC75	14.93 ng/mL
Intra-assay CV%	4.1–7.1%
Inter assay CV%	7.9–17.1%
Dilution recovery (1 + 1 from MRD)	88.5–91.8%
Accepted maximum freeze-thaw	106%
Interference lipid, low/high	104%/104%
Interference hemoglobin, low/high	104%/96%
Interference biotin	86%/89%
Analyte stability (48 h 4 °C/48 h 20 °C)	106%/100%
Accepted maximum freeze/thaw	4 freeze-thaw cycles

### PRO-C12 in serum of patients with solid cancers

**Fig. 2:**
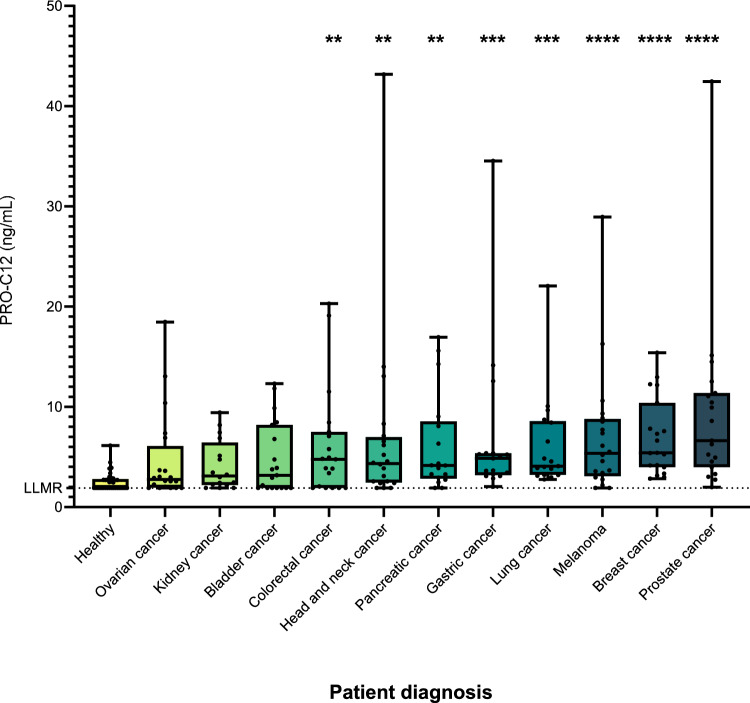
Quantification of PRO-C12 in serum from patients with bladder (19), breast (19), colorectal (19), head and neck (20), kidney (16), lung (17), ovarian (20), pancreatic (17), prostate (19), gastric cancer (17) or melanoma (20) and 33 age-matched healthy controls. PRO-C12 levels are shown as Tukey-style boxplots. For samples with measurements falling below the LLOQ were given the LLOQ value determined during assay validation. Differences in the PRO-C12 levels among groups were analyzed using Kruskal-Wallis test corrected for multiple comparisons using Dunn’s test. **** indicates a *p*-value below 0.0001. *** indicates a *p*-value below 0.001. ** indicates a *p*-value below 0.01. * indicates a *p*-value below 0.05

To investigate the potential use of PRO-C12 in a cancer context, PRO-C12 levels were measured in serum from patients with various cancer types, including bladder (19), breast (19), colorectal (19), head and neck (20), kidney (16), lung (17), ovarian (20), pancreatic (17), prostate (19), gastric cancer (17) or melanoma (20) along with 33 healthy controls (Table 1). PRO-C12 levels were significantly elevated in patients with prostate (*p* < 0.0001), breast (*p* < 0.0001), melanoma (*p* < 0.0001), lung (*p* = 0.0001), gastric (*p* = 0.0004), pancreatic (*p* = 0.0019), head and neck (*p* = 0.0036) and colorectal cancer (*p* = 0.0048) compared to healthy controls (Figure 2), whereas patients with ovarian, kidney and bladder showed similar levels of PRO-C12 compared to healthy controls. When evaluating associations with disease stage, we did not observe any significant differences PRO-C12 (Supplementary Fig. 1). Furthermore, there was a noticeable inter-subject variation of PRO-C12 among patients with cancer when contrasted to healthy subjects.

Regarding diagnostic accuracy based on AUROC calculations, PRO-C12 was good at discriminating cancer patients from healthy controls, in particular patients with breast (AUROC = 0.95), prostate (AUROC = 0.94), lung (AUROC = 0.93), gastric (AUROC = 0.9), melanoma (AUROC = 0.86), pancreatic (AUROC = 0.83) and head and neck cancer (AUROC = 0.79) (Table 3). In summary, these findings indicate elevated levels of PRO-C12 and type XII collagen in serum from patients with different cancer types.

**Table 3:** Area under the ROC curve (AUROC) for the comparison between cancers and controls

Cancer type	AUROC	Cutoff	Sensitivity	Specificity
Breast cancer	0.95	2.91	94.7	81.8
Prostate cancer	0.94	3.01	89.5	84.8
Lung cancer	0.93	2.91	94.1	81.8
Gastric cancer	0.90	3.01	88.2	84.8
Melanoma	0.86	3.38	75	87.9
Pancreatic cancer	0.83	2.91	76.5	81.8
Head & neck cancer	0.79	3.85	60	90.9
Colorectal cancer	0.76	3.01	68.4	84.8
Kidney cancer	0.72	2.91	56.2	81.8
Ovarian cancer	0.68	2.91	50	81.8
Bladder cancer	0.68	2.91	57.9	81.8

### Type XII collagen is expressed by CAFs and NFs

**Fig. 3:**
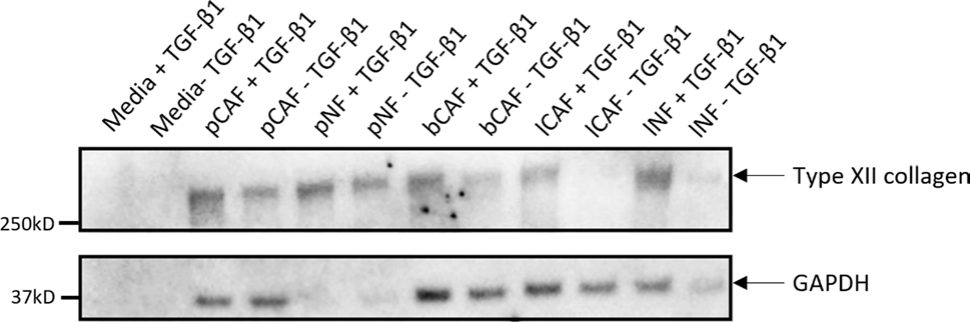
Western blot results of type XII collagen in supernatant from pancreatic CAFs (pCAF), pancreatic NFs (pNF), breast CAFs (bCAF), lung CAFs (lCAF) and lung NFs (lNF) treated and untreated with tgf-β1. A 333kDa fragment corresponding to the full size of type XII collagen was detected together with a 37kDa fragment corresponding to GAPDH The uncropped version of the western blot can be found in the supplementary material as Supplementary Fig. S2

Western blot analysis on the supernatant obtained from different types of CAFs and NFs treated and untreated with TGF-β1 to induce fibrosis revealed the presence of type XII collagen. The observed band, appearing at approximately 333kDa, corresponds to the full-size protein. Although discerning differences can be challenging, our analysis suggests a potentially elevated expression of type XII collagen when fibroblasts are stimulated with TGF-β1, with a notable emphasis on lung derived CAFs and NFs with a 21.1 and 6.5-fold-increase respectively (Figure 3, Supplementary Fig. 2 and Supplementary Table 1).

### *COL12A1* gene expression in TCGA and GTEx databases

**Fig. 4:**
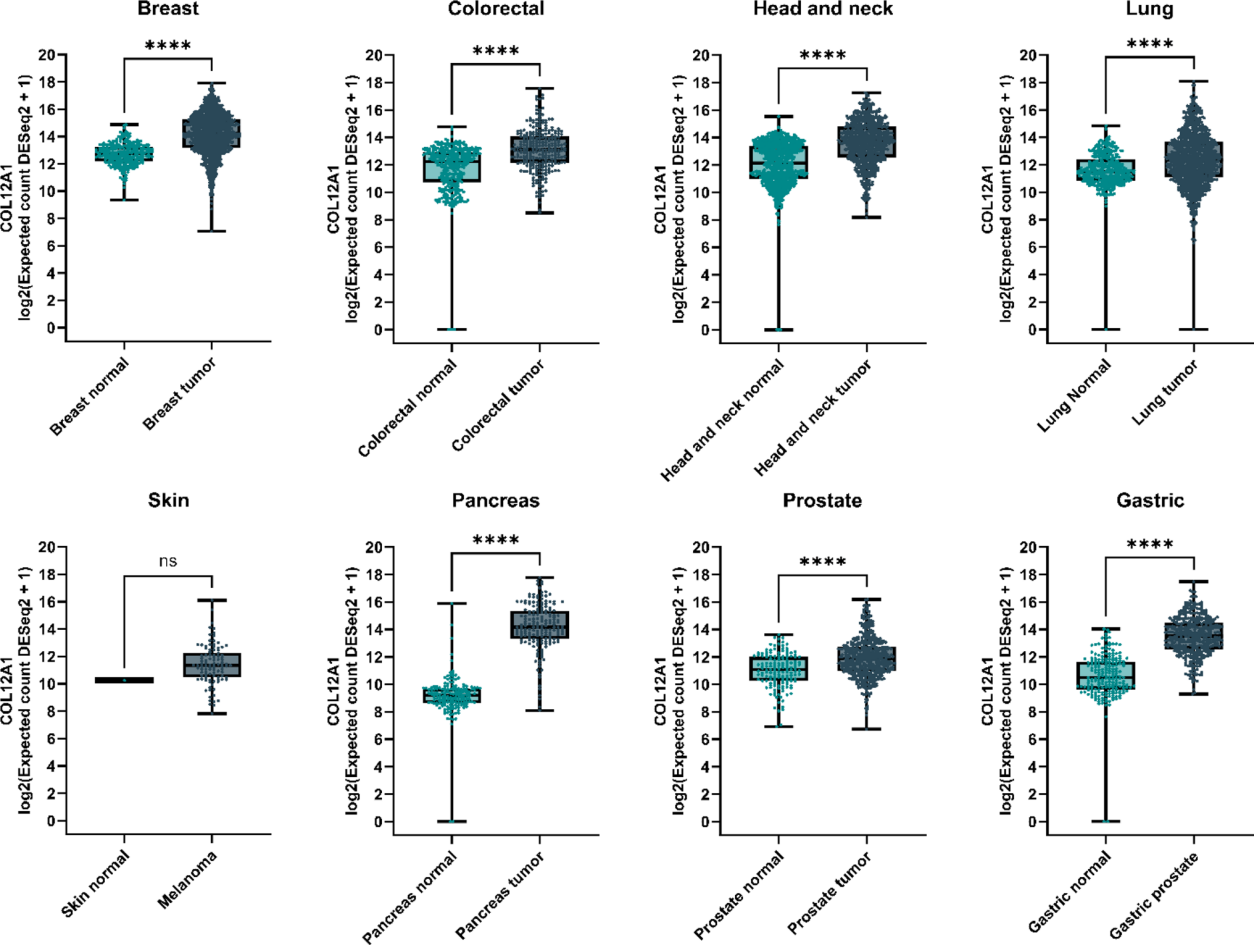
*COL12A1* gene expression obtained from the Cancer Genome Atlas (TCGA) and Genotype-Tissue Expression (GTEx) databases. The normal groups included normal samples from GTEx and tumor-adjacent normal samples TCGA

To validate the relevance of type XII collagen in cancer, we examined the expression levels of *COL12A1* normal and tumor tissue in the specific cancer types were we previously observed increased PRO-C12 levels using publicly accessible data from The Cancer Genome Atlas (TCGA) and Genotype-Tissue Expression (GTEx) initiatives [62, 63]. The normal data set included 2833 normal samples and 4121 tumor samples from the GTEx dataset and tumor-adjacent normal tissue from the TCGA dataset (Table 4).*COL12A1* expression was significantly increased in patients with breast (*p* < 0.0001), colorectal (*p* = <0.0001), head and heck (*p* = <0.0001), lung (*p* = <0.0001), pancreas (*p* = <0.0001), prostate (*p* = <0.0001) and gastric (*p* = <0.0001) cancer (Figure 4). Nonetheless, while the difference did not reach statistical significance, the expression of COL12A1 was higher in patients with melanoma compared to healthy tissue (*p* > 0.05). Further, analysis of cancer stages (Stages I-IV) across various cancer types within the TCGA dataset revealed no significant differences and the expression levels of COL12A1 were consistent across the various cancer stages and types examined (Supplementary Fig. 3). Taking together, these findings are in agreement with our PRO-C12 measurements in circulation.

**Table 4:** Summary of the samples obtained from the Cancer Genome Atlas (TCGA) and Genotype-Tissue Expression (GTEx) databases

Characteristic		
Sample type	*n* (%)	Expression, mean
Breast normal	291 (4.18)	12.7
Breast tumor	1092 (15.7)	14.13
Colorectal normal	348 (5)	11.71
Colorectal tumor	287 (4.13)	13.1
Head and neck normal	709 (10.2)	12
Head and neck tumor	699 (10.05)	13.61
Lung normal	397 (5.71)	11.61
Lung tumor	1091 (15.69)	12.37
Skin normal	556 (8)	11.7
Melanoma	102 (1.47)	11.4
Pancreas normal	171 (2.46)	9.16
Pancreas tumor	178 (2.56)	14.18
Prostate normal	151 (2.17)	11
Prostate tumor	494 (7.1)	11.93
Gastric normal	210 (3.02)	10.6
Gastric tumor	178 (2.56)	13.49

## 4. Discussion

In this study, we developed and validated an ELISA targeting the C-terminus of type XII collagen (PRO-C12) PRO-C12 demonstrated high sensitivity, detecting ng amounts of type XII collagen fragments in serum. The assay exhibited specificity, supported by antibody’s specific binding to the selection peptide, and the accuracy within the intricate serum sample matrix.

PRO-C12 was elevated in serum from patients diagnosed with different cancer types including prostate, breast, melanoma, lung, gastric, pancreatic, head and neck and CRC. PRO-C12 demonstrated the high diagnostic accuracy (by discriminating between healthy donors and patients) with several cancer types, particularly breast cancer (AUROC = 0.947) followed by prostate (AUROC = 0.939), lung (AUROC = 0.925), gastric (AUROC = 0.9), melanoma (AUROC = 0.859), pancreatic (AUROC = 0.829) and head and neck cancer (AUROC = 0.788). Furthermore, when culturing fibroblasts from breast, lung and pancreas we observed that type XII collagen was expressed and induced upon TGF beta treatment. It is noteworthy that *COL12A1* expression has been linked specifically to myofibroblast CAFs (myoCAFs) in pancreatic ductal adenocarcinoma (PDAC) and correlated with poor survival [8]. To corroborate our results obtained measuring PRO-C12, we employed *COL12A1* gene expression data from the TCGA and GTEx databases and confirmed the increased expression of type XII collagen in those specific groups of patients. In humans, *COL12A1* is expressed during the developmental stage in mesenchymal tissues whereas in adults its presence is limited to basement membrane (BS) zones and fascia of muscle, kidney and dermis [64]. In cancer tissue, type XII collagen has been found in the invasive front of CRC metastasis and in the stroma of breast invasive ductal carcinoma (IDC) [41, 65].

Interestingly, PRO-C12 had the highest diagnostic accuracy and inter-patients variation in prostate cancer. This is in contrast to other FACIT collagens such us collagen types XIX, XX and XXII measured in the same cohort of patients [13, 14, 66]. Prostate cancer frequently metastasizes to bone but the mechanisms driving metastasis to the osteoblastic niche remain unclear even though the ECM possibly plays a significant role in facilitating this process [67].

Other ECM proteins such as tenascin-C, expressed in the nervous system during bone development and essential for wound healing in adults has been proposed as key hallmark of reactive stroma response in prostate cancer, promoting metastasis to bone through integrin α9β1. In vitro studies suggest tenascin-C induces type XII collagen production, potentially leading to elevated PRO-C12 levels [68–73]. In this context, PRO-C12 could be used as a tool to monitor the efficacy of anticancer treatments that disrupt the α9 integrin-tenascin-C interaction and improve the prognosis of patients with metastatic prostate cancer [73].

Type XII collagen has been proposed as potential prognosis biomarker for predicting clinical outcomes and as anti-cancer treatment target in several tumor types. For instance, a study reported that in patients with gastric cancer type XII collagen expression was increased both at the mRNA and protein levels and that in was mainly expressed by the CAFs in surrounding the tumor [74]. Furthermore, overexpression of type XII collagen correlated with advanced TNM stage, metastasis and worse clinical outcomes [74]. Even though the molecular mechanisms underlying type XII in gastric cancer have not been fully elucidated, some studies suggest that IDO1 and type XII collagen together could induce gastric metastasis [43]. Knocking down both IDO1 and COL12A1 in gastric cancer cells (SGC-7901) led to greater ERK phosphorylation inhibition and reduced cell migration. Integrin β1 overexpression reversed MAPK pathway inhibition, indicating activation of the MAPK pathway by both IDO1 and type XII collagen via integrin β1. Overall, they propose a mechanism where type XII collagen could facilitate interactions between collagen fibrils and integrin β1, leading to ERK phosphorylation in gastric cancer cells and tumor dissemination. In this context, IDO1 and type XII collagen might be promising targets to treat gastric cancer, and PRO-C12 could help to assess which patients respond to treatment [43].

Remarkably, our study revealed elevated levels of PRO-C12 in patients diagnosed with PDAC. This observation aligns with previous research and could be indicative of CAF activation. CAFs are known contributors to the tumor microenvironment and have been specifically identified as expressing type XII collagen. In our study, we detected the presence of type XII collagen in the supernatant of both CAFs and NFs. Notably, upon treatment of fibroblasts with TGF-b1 which induces tumor fibrosis and collagen synthesis, we observed a discernible trend towards and increased production of type XII collagen. This suggests a potential regulatory role of TGF-b1 in the synthesis of type XII collagen by fibroblasts providing insight into the dynamic nature of extracellular matrix remodeling in response to TGF-β1 stimulation. In earlier investigations involving patients with PDAC, higher expression of type XII collagen was detected in disease stages III and IV compared to stages I and II and correlated with poor prognosis [75]. When analyzing genetic alterations in PDAC tissues qPCR proved that type XII collagen mainly derived from CAFs and not by tumor cells. Moreover, type XII collagen expression exhibited a positive correlation with genes linked to fibroblast activation such as FAP, vimentin, α-SMA and ACTA2. Additionally, pathway correlation analyses revealed a significant association between type XII collagen expression and processes such as collagen formation, ECM-related genes, the TGF-β pathway, and the inflammation signature and interestingly, knockdown of *COL12A1* resulted in inhibition of CAF invasion and reduction of CAF associated biomarker expression. This interconnected relationship underscores the potential role of type XII collagen in influencing various molecular pathways associated with the intricate dynamics of the ECM and inflammatory responses [75]. Moreover, type XII collagen is expressed not only by CAFs but also by CRC cells in the desmoplastic front, and we could speculate that beyond its potential role as CAF biomarker, may also an indicator for cancer cells in the invasive front [41]. In studies in breast cancer, it was confirmed that CAFs deposit type XII collagen in the TME. Additionally, they observed that the heightened stiffness attributed to increases type XII collagen deposition, induced a change of phenotype of normal fibroblasts into myofibroblasts, thus facilitating the formation of a pro-invasive TME that facilitate tumor invasion [49]. As type XII collagen deposition increases as the tumor develops PRO-C12 may serve as a biomarker of early dissemination, and it could help to identify those patients with breast cancer that are at high risk of tumor metastasis.

Type XII collagen belongs to the FACIT family and plays a role the fibril assembly of fibrillar collagens like type I and III. Type XII collagen contains a domain that binds to fibrillar collagens and contributes to the interaction and organization of fibril structures, providing additional stabilization. Recent studies have highlighted how desmoplastic lesions are characterized by chaotic collagenous deposition surrounding the tumor and that this altered ECM organization differs from normal structure in healthy tissue. We could speculate that CAFs recruited by cancer cells would attempt the reorganization of the ECM surrounding the tumor through various mechanisms, potentially involving production of type XII collagen that would be reflected by high levels of PRO-C12 in circulation. Nevertheless, further experiments are imperative to elucidate the mechanisms behind its secretion and its precise role in tumor progression.

The PRO-C12 ELISA was designed to target the C-terminus of type XII collagen, but the cleavage process releasing the protein fragment into the bloodstream remains unclear. We could argue that the complete type XII collagen protein would remain in the ECM organizing fibrillar collagens and that the fragments found in circulation might have lost their anchoring properties and are result from excessive ECM remodeling occurring in the TME. Increased levels of PRO-C12 in patients with cancer could reflect the excessive production of type XII collagen by CAFs and tumor cells in the invasive front as discussed previously. Nevertheless, the intricacies of this process remain poorly described, and more investigations to clarify the processing and cleavage of type XII collagen are warranted.

While our study provides valuable insights, it is essential to consider its limitations. The number of patients in each of the cancer groups was small, heightening the probability of introducing bias and generating inaccurate positive results. Overall, in the cancer group we observed a higher spread of PRO-C12 levels among the patients compared to the healthy donors as well as large fold-increases in the biomarker levels. Lack of clinical data in the cohort constrained the analysis to a comparison of PRO-C12 levels between groups but with minimal regard for their clinical implications emphasizing the necessity to validate these results in a cohort that is more thoroughly characterized. Additionally, it would be interesting to evaluate if increasing PRO-C12 levels correlate with tumor stage and if it can be used to predict which patients are more prone to metastasis. Nevertheless, this is our first study exploring the biomarker potential of type XII collagen in cancer in its quantification in a non-invasive manner in serum samples. Based on this preliminary data we believe that PRO-C12 is a biomarker that could have a great impact in cancer patients, and additional research into the biology of PRO-C12 is needed.

## Supplementary Information

Below is the link to the electronic supplementary material.

Supplementary file1 (DOCX 676 kb)

## Abbreviations

ECM: Extracellular Matrix
TME: tumor microenvironment
CAF: cancer associated fibroblast
NF: normal fibroblast
FACITs: fibrillar-associated collagens with interrupted triple helices
COL: collagenous
NC: non-collagenous
KLH: keyhole limpet hemocyanin
LLOQ: lower limit of quantification
ULOQ: upper limit of quantification
ELISA: enzyme-linked immunosorbent assay
PBS: phosphate-buffered saline
BSA: bovine serum albumin
RPM: revolutions per minute
TMB: 3,3’,5,5’-Tetramethylbenzidine
HRP: horseradish peroxidase
AUROC: area under the receiver operating characteristic
TGF-β: transforming growth factor beta
mEDS: myopathic type Ehlers-Danlos syndrome
CRC: colorectal cancer
PDAC: pancreatic ductal adenocarcinoma
CV: coefficient of variation
LLMR: lower limit of measurement range
ULMR: upper limit of measurement range
LLOB: lower limit of detection
FBS: fetal bovine serum
TBST: Tris-buffered saline with 0.1% Tween® 20 detergent
TCGA: The Cancer Genome Atlas Program
GTEx: Genotype-Tissue Expression
IDO1: Indoleamine 2,3-Dioxygenase 1
ERK: extracellular signal-regulated kinase
MAPK: Mitogen-activated protein kinase
FAP: fibroblast activation protein
α-SMA: alpha smooth muscle actin
ACTA2: actin alpha 2
