# Supplementary material for: Type XII collagen is elevated in serum from patients with solid tumors: a non-invasive biomarker of activated fibroblasts
Source: Clin Exp Med. 2024 Jul 24;24(1):166. doi: 10.1007/s10238-024-01431-y (PMC11269340; doi:10.1007/s10238-024-01431-y)
Supplement: Supplementary file 1 — Supplementary file1 (DOCX 676 kb) [file 10238_2024_1431_MOESM1_ESM.docx]

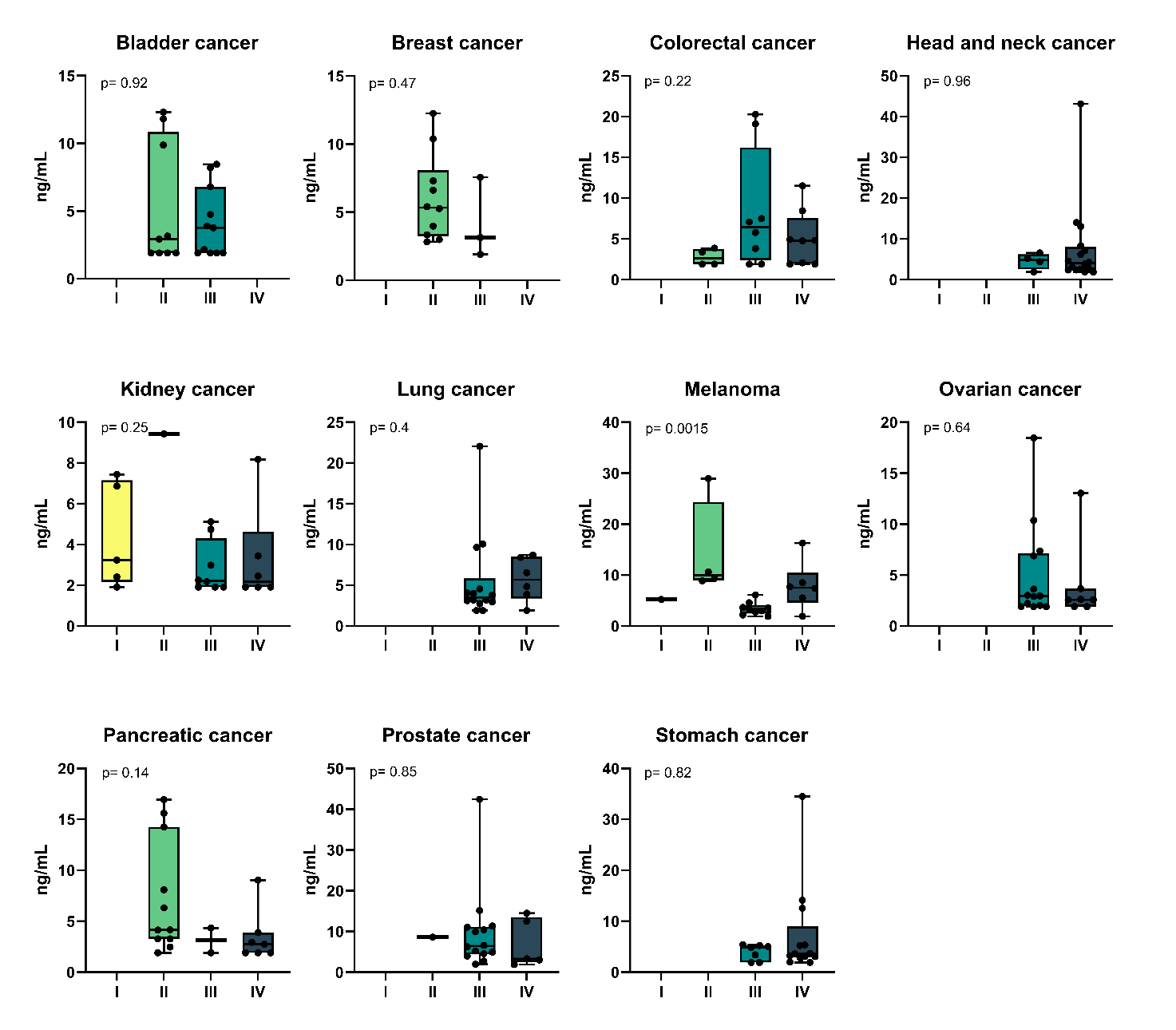


**Supplementary figure 1** PRO-C12 levels according to cancer stage. Biomarker levels were shown as Tukey-style boxplots. Differences in PRO-C12 according to cancer stage in each cancer type was evaluated by Kruskal-Wallis


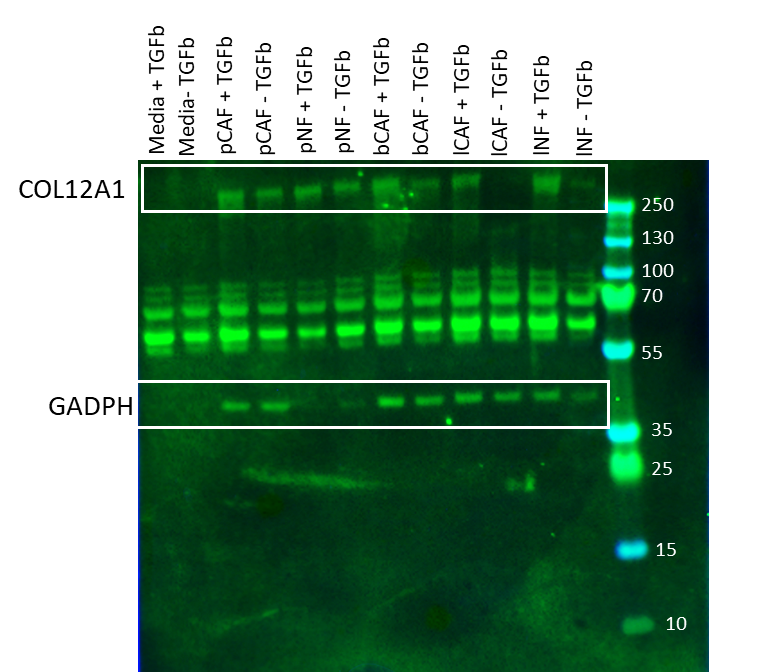


**Supplementary figure 2** Uncropped western blot results of type XII collagen in supernatant with the PRO-C12 antibody and GAPDH as loading control


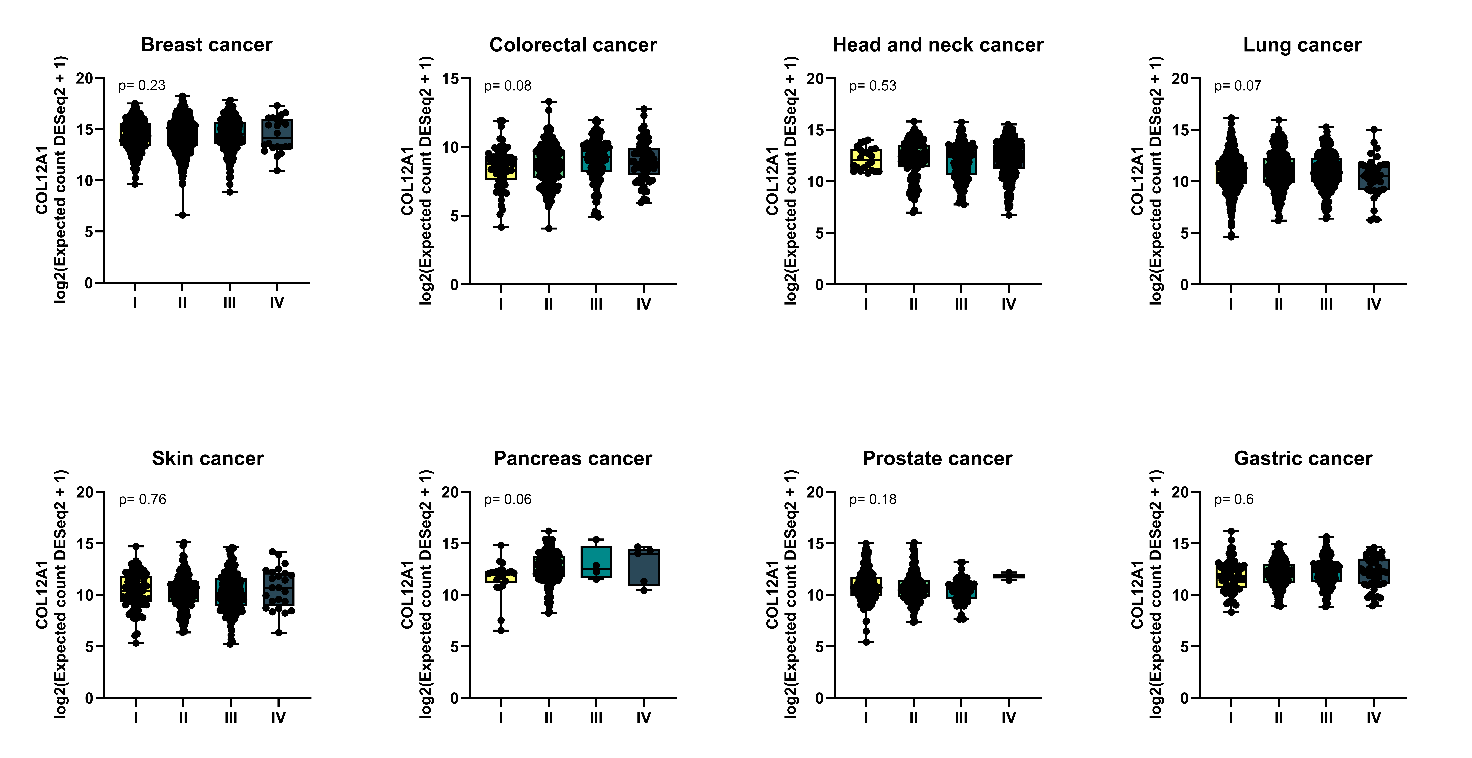


**Supplementary figure 3** COL12A1 gene expression obtained from the Cancer Genome Atlas (TCGA) database according to cancer stage.

**Supplementary table 1** Fold change in type XII collagen expression from Western blot analysis in various cell types, comparing stimulated (TGF-β) and non-stimulated conditions. Fold changes are normalized to the loading control (GAPDH).

| **Cell type** | **Fold-change** |
| --- | --- |
| pCAF | 1.65 |
| pNF | 1.87 |
| bCAF | 2.21 |
| lCAF | 21.12 |
| lNF | 6.51 |
